# Necrotizing fasciitis secondary to lake water inoculation with *Aeromonas sobria*: A case report

**DOI:** 10.1097/MD.0000000000024981

**Published:** 2021-03-12

**Authors:** Lauren E. Hutchinson, Jacob D. Franke, Brian A. Mailey

**Affiliations:** a Institute for Plastic Surgery, Southern Illinois University School of Medicine; b Southern Illinois University School of Medicine, Springfield, IL.

**Keywords:** *Aeromonas sobria*, lake water infection, necrotizing fasciitis

## Abstract

**Rationale:**

Necrotizing fasciitis (NF) is a rapidly progressing bacterial soft tissue infection with a high mortality rate. It is characterized by significant soft tissue destruction with associated sepsis. The mainstay of treatment is coverage with appropriate broad-spectrum antibiotic therapy and emergent surgical debridement.

**Patient concerns:**

A previously healthy 66-year-old female presented with a deep laceration to her right, posterior calf with subsequent contamination with lake water. After the wound was irrigated and closed, the patient developed NF.

**Diagnosis:**

Laceration of the right lower extremity complicated by NF secondary to *Aeromonas sobria.*

**Interventions:**

The patient underwent emergent surgical debridements with intravenous broad-spectrum antibiotics and negative pressure wound therapy. The lower extremity was reconstructed with split-thickness skin grafts.

**Outcomes:**

The patient's initial penetrating trauma was closed in the emergency room, and the patient was discharged home with antibiotics. She returned the next day with unstable vitals and was admitted to the intensive care unit. Her condition continued to deteriorate, and she underwent serial surgical debridements. Her condition improved and was discharged home after 13 days in the hospital.

**Lessons Learned:**

Close monitoring for NF is important for tissue infections sustained in aquatic environments. Timely identification and surgical management of NF increases overall survival.

## 1 Introduction

Necrotizing fasciitis (NF) is a rapidly progressing bacterial soft tissue infection that carries a high mortality rate. Lifesaving initial treatment includes broad-spectrum antibiotic therapy and emergent surgical debridement.^[1,2]^ The incidence of NF ranges from 0.3 to 5 cases per 100,000 people/yr and can have a fulminant course with an associated mortality rate between 40% and 60%.^[3]^ While NF is uncommon, certain patient populations are more likely to be affected. Patients at higher risk for NF include those with an immunocompromised status, uncontrolled diabetes mellitus, liver disease, alcoholism, end-stage renal disease, malignancy, and intravenous drug abusers.^[4,5]^

NF produces significant soft tissue destruction with associated systemic symptoms and sepsis. Clinical history often involves a penetrating trauma to the area involved, although no history of trauma may be present.^[6,7]^ Physical exam findings usually demonstrate fever, soft-tissue edema, erythema, and overlying skin changes including bullae formation, grey-blue discoloration, and tissue necrosis. These are also accompanied by severe pain disproportional to exam findings which is often the clearest indicator of NF in the setting of the other findings.^[7]^ The patient will also display systemic signs of severe infection including, hypotension, tachycardia, leukocytosis, elevated creatinine, hyponatremia, hyperglycemia, C-reactive protein, and lactic acid.

NF is categorized into 2 microbiological categories. Type 1: polymicrobial; and Type 2: monomicrobial infection. Polymicrobial infections are caused by both aerobic and anaerobic bacteria. Monomicrobial infections result most commonly from infection with group A Streptococcus, followed by *Clostridium perfringes* and methicillin-resistant *Staphylococcus aureus*. Other less commonly involved pathogens include *Aeromonas hydrophilia* and *Vibrio vulnificus*.^[8]^

*Aeromonas* is an oxidase-producing gram-negative bacillus that is abundant in freshwater, saltwater, and soil. It is also found in various foods including dairy products, meat, and fresh vegetables. Three species of *Aeromonas* are found to have clinical implications in humans including *hydrophilia*, *veronni biovar sobria*, and *caviae*. Infections from these *Aeromonas* strains can result in gastrointestinal illness, hepatobiliary infections, pneumonia, meningitis, bacteremia, and soft-tissue infections.^[9,10]^ Soft tissue infections from *Aeromonas sobria* are extremely rare and can occur following trauma to the skin that was sustained in an aquatic environment. These necrotizing infections have previously been reported to involve individuals with predisposing factors of poorly-controlled diabetes mellitus, hematological malignancies, or immunocompromised status.^[11,12]^ Herein, we present a unique case of a healthy, nondiabetic female who developed NF secondary to contamination of an open wound with lake water.

## 2 Case presentation

The patient presented herein has agreed and given her consent for her clinical details and images to be used for scientific publication; formal institutional review board approval was not required for consented patient case reports at our institution.

A 66-year-old Caucasian female with a medical history significant for hypertension, asthma, and chronic adrenal insufficiency on 5 mg of prednisone daily developed NF 24-hours after sustaining a penetrating trauma to her right lower extremity. The patient fell from a dock while disembarking from a boat. She sustained a deep laceration of her right posterior calf immediately before falling and completely submerging the laceration in lake water. She presented to the emergency department for evaluation and treatment the same evening.

**Figure 1:**
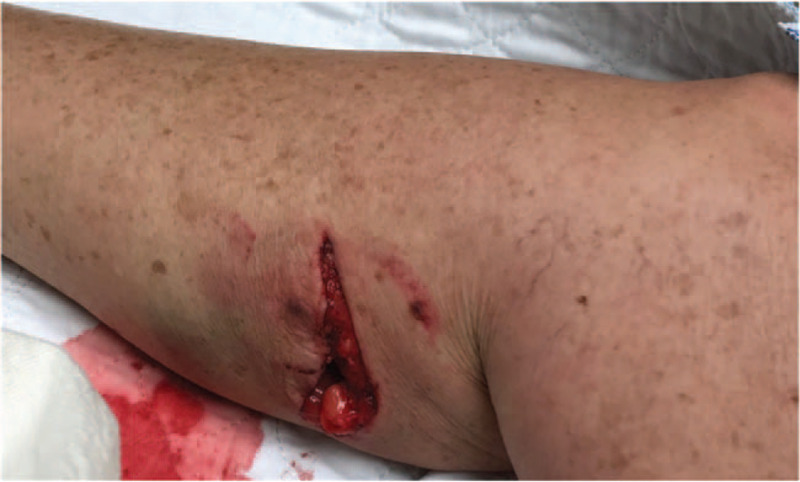
Lower extremity laceration at time of presentation to emergency department.

On examination, she sustained an 8 cm laceration on her right, posterior calf that tracked to the fascia overlying the gastrocnemius (Fig. 1). X-rays were negative for fractures or retained foreign material. The wound was irrigated with betadine and normal saline at the bedside in the emergency department and then repaired in a 2-layer closure by the plastic surgical team. She was discharged with 10-day course of Augmentin.

The following morning, approximately 14 hours after discharge, she represented to the emergency department with subjective fevers, several episodes of emesis, malaise, anorexia, and worsening pain on her injured calf at the site of laceration repair. She was unable to take any doses of the prescribed Augmentin due to nausea and emesis. On presentation, she was tachycardic with heart rates in the 110 seconds, hypotensive with systolic blood pressures in the 80 seconds, a respiratory rate of 18, and a temperature of 36.6°C. Laboratory evaluation revealed a leukocytosis with left shift, white blood cells 20.4 (80% polymorphonuclear leukocytes), lactic acidosis of 2.8, procalcitonin 28.4, blood urea nitrogen (BUN) 24, creatinine 0.9, glucose 140, creatine kinase 253, and C-reactive protein 36.2. Duplex ultrasonography was obtained, and plain film x-rays were repeated and both negative. On clinical evaluation by the emergency and internal medicine physicians, her right calf had minimal erythema, no crepitus or fluctuance but was tender to palpation. She was diagnosed with septic shock secondary to right leg cellulitis and admitted to the intensive care unit (ICU) for resuscitation, blood pressure support, and started on broad-spectrum antibiotics with intravenous vancomycin and piperacillin/tazobactam.

**Figure 2:**
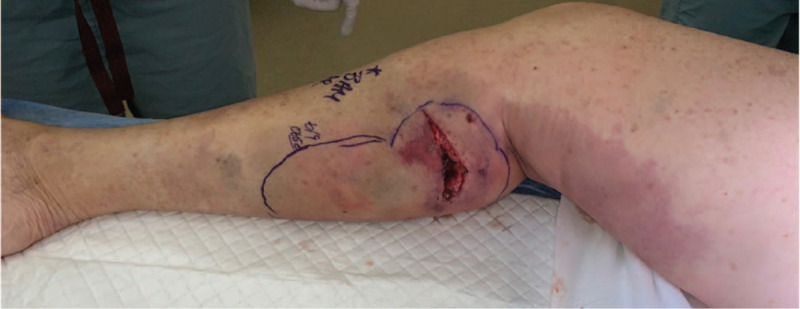
Appearance of lower extremity on hospital day 1.

Overnight her clinical course continued to deteriorate. Her vasopressor requirements increased, her leukocytosis persisted, and she demonstrated signs of end organ hypoperfusion with a rising creatinine and BUN from 0.9 to 1.3 and 24 to 27, respectively. The surgical service that originally repaired her laceration was then consulted. On physical examination, her right calf was extremely tender to palpation and the skin surrounding the repaired laceration had purple discoloration. Sutures were removed at the bedside, and there was significant undermining of the subcutaneous tissue along the posterior compartment fascia, devitalized fat, and dishwater murky fluid was present. Based on these findings as along with her clinical picture a diagnosis of NF was made. She was taken emergently to the operating room (OR) for excisional debridement of her right lower extremity (Fig. 2).

Intraoperatively, a 15 cm × 13 cm area of devitalized tissue was excised including skin, subcutaneous tissue, and muscle fascia from her right calf. Tissue samples were obtained and sent for culture and sensitivity. Postoperatively, she returned to the ICU for further monitoring. Infectious disease assisted with managing her antimicrobial therapy and added intravenous Clindamycin for its antitoxin effects and ciprofloxacin for Aeromonas coverage given the history of lake water exposure on top of the vancomycin and piperacillin/tazobactam.

**Figure 3:**
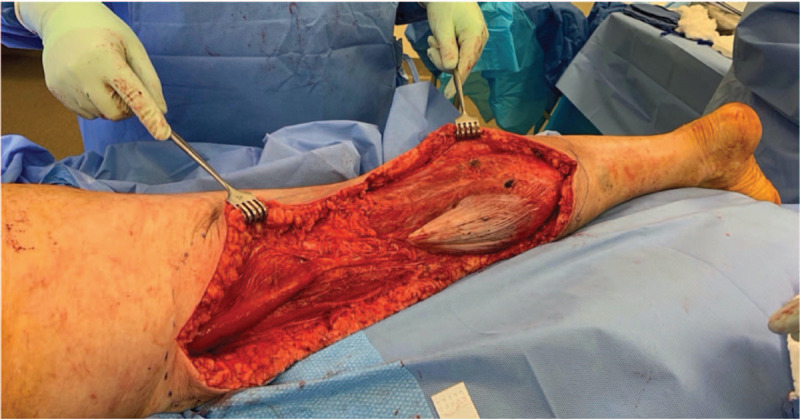
Lower extremity after second surgical debridement.

Given the rapid onset of NF after her injury and concern for possible further progression, serial exams of her lower extremity were performed throughout the day. Later that day, progression of the infection occurred. Her lactate rose to 4.1 and continued vasopressor support was required. The patient was taken back to the OR for an additional round of debridement that same day. The infection had spread proximally and distally from the previous debridement site. A 31 cm × 19 cm area of her calf and posterior thigh were excised down to muscle (Fig. 3).

Postoperative day 1 (POD1) her clinical condition dramatically improved. Her hemodynamic instability resolved, and she was taken off vasopressors. The white blood cells decreased to 17.4 and creatinine trended down to 1.1. On exam, her pain significantly improved. On POD2 the intraoperative tissue cultures results showed pansensitive *Aeromonas veronii biovar sobria*. Vancomycin was discontinued, and she was continued on piperacillin/tazobactam, clindamycin, and ciprofloxacin until the negative anaerobic cultures resulted. On POD3 (hospital day 4) her condition had improved, and she was downgraded from the ICU.

She underwent 4 serial debridements of her right lower extremity during her hospitalization. Additional debridements occurred on hospital days 4 and 7 to ensure all nonviable tissue had been excised. After the final debridement, her wound was temporized with negative pressure wound therapy to ensure a healthy wound bed with adequate granulation tissue present for reconstruction. She was discharged home with negative pressure wound therapy on hospital day 13. ACell (MatriStem) porcine bladder submucosa matrix was used to incite development of granulation tissue at her final debridement.

**Figure 4:**
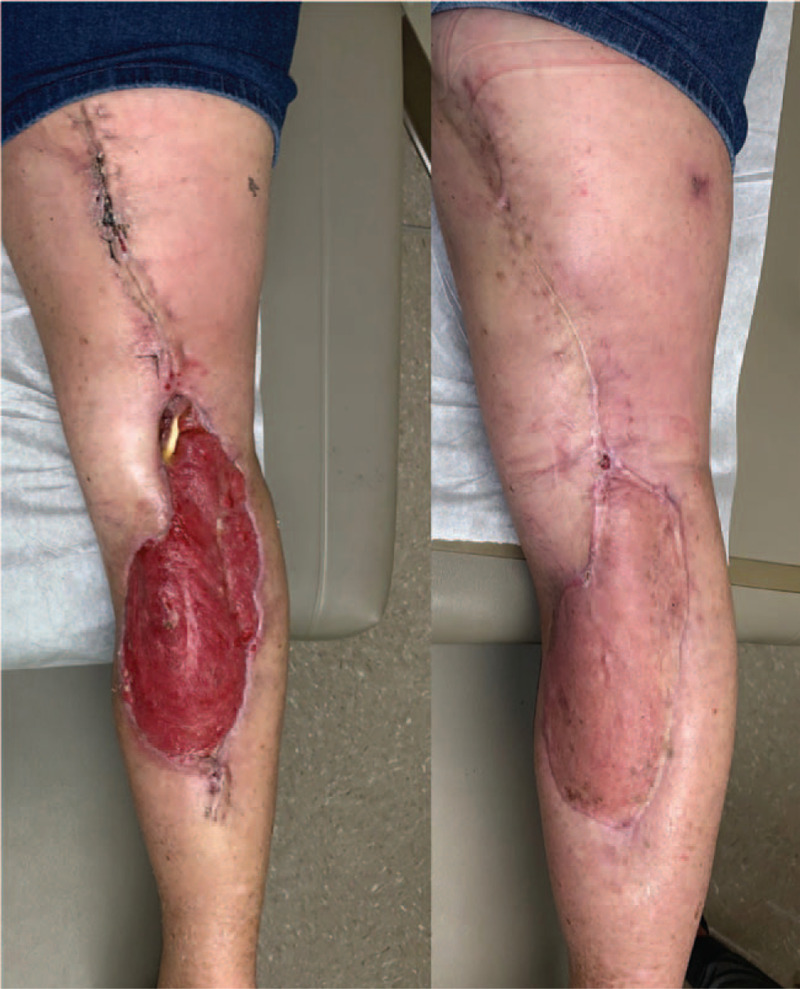
Following negative pressure wound therapy; 2 mo following split-thickness skin grafting reconstruction.

One month after the final debridement, she was taken to the OR for definite reconstruction with split-thickness skin grafting. At 2 months status post-reconstruction, her wounds have completely healed, and she has returned to her usual activities (Fig. 4).

## 3 Discussion

NF is uncommonly caused by Aeromonas bacteria. Previous data reports the overall incidence from infection from *Aeromonas* was 10.6 per 1 million people with wound infections accounting for 0.7 per 1 million people.^[13]^ Aeromonas soft tissue infections generally occur following trauma to the skin which is then exposed to water. The most common location for an Aeromonas infection is the lower extremity which was consistent with our patient.^[14]^ Previously reported literature demonstrates NF infections caused by *Aeromonas* species are associated with higher mortality than other pathogens and usually present 72 hours after inoculation.^[5]^ Additionally, Spadaro et al's review of the literature suggests that *A sobria* produces several virulent factors that can lead to septic shock. In fact, of the cases reported in the literature, there was a >90% mortality rate when the pathogen was *A sobria*.^[11]^

Despite the odds, our patient survived her severe necrotizing infection with associated sepsis and has completely healed her wounds at this time. We attribute this to early patient presentation following laceration repair as well as early surgical debridement. There was also a low threshold to return to the OR for serial debridements which also likely improved her outcome. In order to reduce morbidity and mortality from NF infections, it is extremely important to identify the infection early and treat appropriately with antibiotics and emergent surgical debridement. Patients with NF commonly present with local pain, erythema, and swelling. Vital sign abnormalities can include tachycardia, hypotension, and tachypnea.^[8]^ Wong et al found that a delay in the initial surgical debridement beyond 24 hours of the onset of symptoms is correlated with increased mortality.^[15]^ Further complicating early interventions, studies have demonstrated that patients with an immunocompromised status are less likely to have an elevated white count and more likely to have a delay in diagnosis and first surgical debridement of NF.^[16]^

Acute open wounds exposed to aquatic environments should raise clinical suspicion for possible *Aeromonas* infections, especially since it has higher mortality than other pathogens associated with NF. Despite previous cases primarily occurring in individuals with comorbidities of either uncontrolled diabetes mellitus, malignancies, or immunocompromised status, necrotizing infections from *A sobria* may also occur in healthy individuals.

## 4 Conclusion

This is a case report of a rare NF infection resulting from an *A sobria* infection in a healthy patient. When a patient sustains an injury in a waterborne environment and presents in septic shock, it is imperative to have a high clinical suspicion for possible *Aeromonas* infection. The patient should be treated immediately with a fluoroquinolone in addition to appropriate broad-spectrum antibiotic therapy and should have an emergent surgical consultation and debridement.

## Author contributions

**Writing – original draft:** Lauren E. Hutchinson, Brian A. Mailey.

**Writing – review & editing:** Lauren E. Hutchinson, Jacob D. Franke, Brian A. Mailey.
